# Anodic Polymerization of Phenylphenols in Methyl Isobutyl Ketone and Mesityl Oxide: Incorporation of a Cavitand into the Layers Formed for Sensing Phenols in Organic Media

**DOI:** 10.3390/molecules27175366

**Published:** 2022-08-23

**Authors:** László Kiss, Zoltán Nagymihály, Péter Szabó, László Kollár, Sándor Kunsági-Máté

**Affiliations:** 1 Department of Organic and Medicinal Chemistry, University of Pécs, Honvéd Street 1, H 7624 Pécs, Hungary; 2 János Szentágothai Research Center, Ifjúság útja 20, H 7624 Pécs, Hungary; 3 Environmental Analytical and Geoanalytical Research Group, Szentágothai Research Center, Ifjúság útja 20, H 7624 Pécs, Hungary

**Keywords:** phenylphenols, methyl isobutyl ketone, mesityl oxide, cavitand

## Abstract

The electropolymerization of three phenylphenol isomers was studied in methyl isobutyl ketone and mesityl oxide, and the remarkable differences highlighted the importance of the carbon–carbon double bond in mesityl oxide. In the case of each substrate, a brownish deposit formed during the electrooxidation. The obvious difference between the polymers formed from the two solvents was recognized via voltammetric signal enhancement of 4-methoxyphenol and 4-chlorophenol, and it was only observed in the case of mesityl oxide. The experiments highlighted that incorporation of a cavitand with biphenyl groups on the upper rim of the polymers of phenylphenols improved the results to a small extent. The cavitand was, itself, electroactive without any fouling effect. As 2-phenylphenol is by far the cheapest of the three isomers, a cavitand was incorporated into its polymer, which was exploited to solve analytical problems while mesityl oxide was used as solvent. Useful quantifications were achieved in organic solvents; however, it failed under aqueous conditions due to the high hydrophobicity of the deposit. Application of differential pulse voltammetry for 4-methoxyphenol and 4-chlorophenol gave detection limits of 9.28 and 50.8 μM in acetonitrile, respectively. This procedure resulted in the immobilization of cavitand derivatives onto the electrode’s surface, and the layer formed offered selective sensing of phenols by electrochemical methods.

## 1. Introduction

Phenylphenols are very attractive phenol derivatives, especially 2-phenylphenol due to the fact of its outgoing practical importance in the food industry (detection in citrus fruits) [1,2,3]. On the other hand, they are important intermediates for organic synthesis, and they are susceptible to oxidative polymerization as is usually characteristic of phenols. Thus, electrochemical study of them in uncommon solvents was the topic of this work. Phenols usually undergo formation of a radical during anodic polymerization, and the prepared deposits have favorable properties exploited in many areas. Herein, the analytical usefulness of poly(phenylphenols) was estimated.

Cavitands are an important class of macromolecules with excellent binding properties, especially in their cavity. As a matter of fact, they are cyclic oligomers of aromatic monomers predominantly consisting of four members interconnected by methylene bridges. This cavity is usually substituted with organic substituents on each member on the lower and/or upper rim, making their binding capabilities more selective. The interaction of organic molecules with molecular capsules is a hot topic of research, as selective binding of certain molecules is possible in their cavity. In the cavity of larger host molecules of cavitands, not only can neutral guest molecules be accommodated but organic ions [4,5] and, moreover, metal ions [6] as well. Predominantly, weak *π–π* interactions are responsible for their binding capability, originating from the delocalized electron system of the aromatic moieties which can surround the guest species. This is the reason why the skeleton of all cavitands also show high affinity to electron-poor aromatics. Therefore, sensing methods based on photoluminescence and electrochemical techniques have widespread application [7,8,9,10,11].

Numerous cavitands have promising properties in electrochemical applications, whether they are electroinactive or if they are electroactive. The favorable binding properties of redox inactive resorcinarenes can be utilized by their incorporation into carbon paste electrodes due to the fact of their accumulation close to the electrode’s surface [12]. In the majority of examples, the cavity skeleton of resorcinarenes is electrochemically inactive and the side chains on their rims have redox properties. Usually, weak electronic communication occurs among them, especially if they remain neutral. In contrast, reports can be found where the appearance of more voltammetric peaks were observed [13].

Earlier studies have pointed out that such remarkable differences come from the fact that either methyl isobutyl ketone (MIBK) or mesityl oxide (MO) are used as a solvent in electropolymerization reactions of organic compounds susceptible to this process [14,15]. However, these solvents differ from each other only in a carbon–carbon double bond; therefore, copolymerization takes place when mesityl oxide is used as a solvent, as the electrochemically generated radicals can undergo saturation reactions, thus altering the structural properties of the formed polymer. Therefore, in the present study, this was one of the selected solvents used to investigate anodic polymerization reactions of phenylphenol monomers and to prepare modifying layers on an electrode. This procedure resulted in the immobilization of cavitand derivatives onto the electrode’s surface, and the layer formed offered selective sensing of phenols by electrochemical methods.

## 2. Materials and Methods

**Figure 1:**
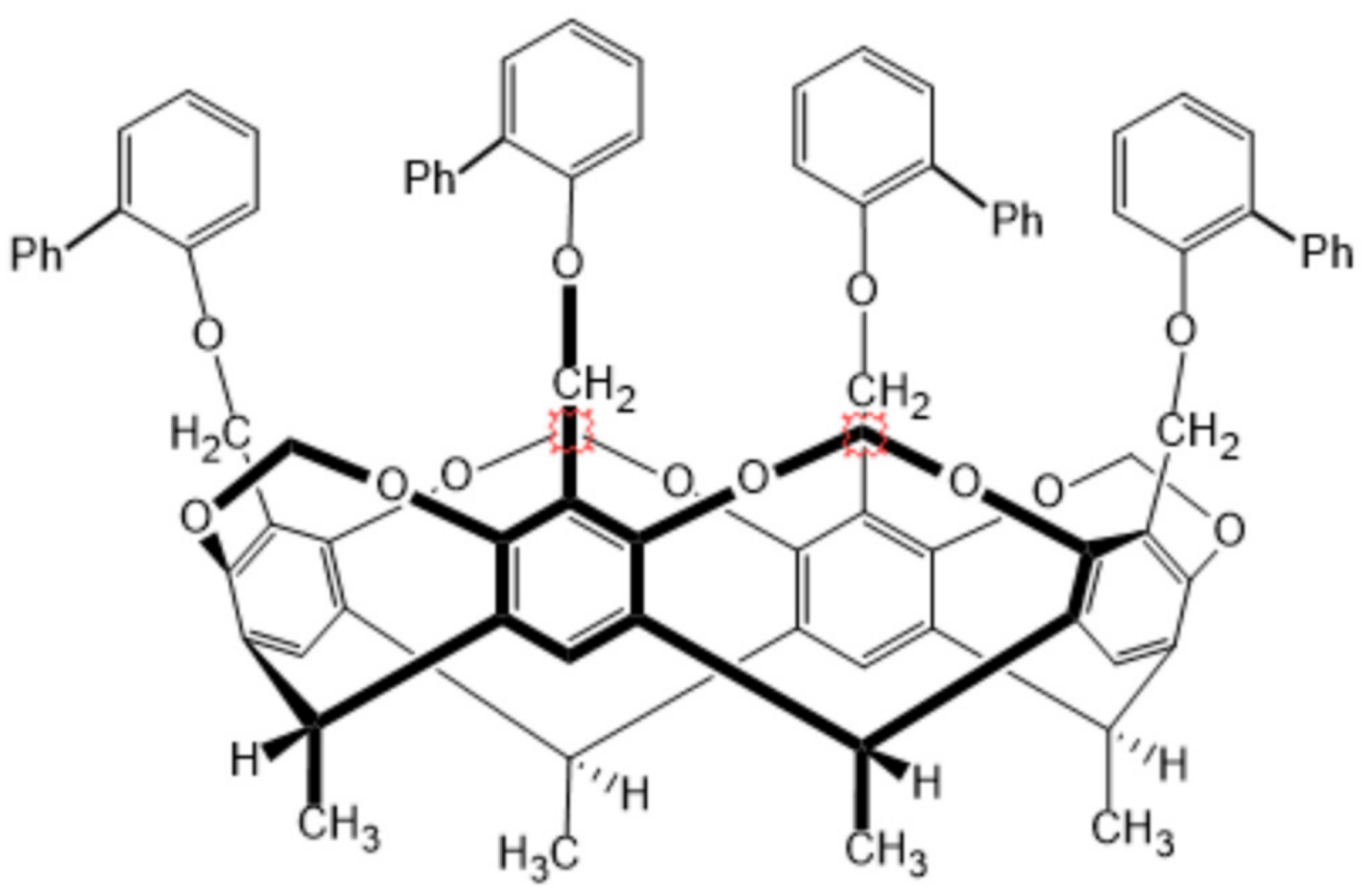
Structural formula of the studied cavitand (**1**).

The solutes investigated in this study were obtained from Merck, and analytical reagent grade solvents were used to prepare the solutions. The cavitand possessing 2-phenylphenoxy moieties on the upper rim (Figure 1) was prepared as described for similar host compounds [16]. The supporting electrolyte in the organic solvents was tetrabutylammonium perchlorate (TBAP), and in aqueous solvents 0.1 M at pH = 7 in phosphate buffer was used.

All voltammetric measurements were carried out with a Dropsens Potentiostat (Spain) connecting to it a three-electrode cell. For the working electrode, a 1 mm diameter platinum disc electrode was used to seal in the polyether ether ketone (PEEK). A platinum rod served as the counter and a silver wire as the reference electrode. A saturated calomel electrode was used as the reference in the aqueous solutions. Before all experiments, the surface of the platinum disc was cleaned by polishing with alumina using a polishing cloth. This was followed by thorough washing with deionized water and, finally, dried by rinsing with dry acetone.

Surface images of the samples were captured with a Jeol JSM-IT500HR (Jeol, Tokyo, Japan) scanning electron microscope (SEM) using the secondary electron mode and a 30 kV acceleration voltage. The samples were coated with gold with a Jeol JFC-1300 auto fine coater (Jeol, Tokyo, Japan). The indium–tin oxide (ITO)-coated glass slides used for the microscopic experiments had an 8–12 Ω/sq surface resistance. Their surfaces were cleaned before use in an ultrasonic bath by immersion in ultrapure water to remove the physically adsorbed particles and then thoroughly washed with dry acetone.

### Synthesis of the Cavitand (***1***)

First, 3.00 mmol (511 mg) of 2-phenylphenol and 6.00 mmol of K_2_CO_3_ (830 mg) were dissolved in 15 mL of dimethyl sulfoxide in a 50 mL Schlenk flask under an argon stream. The mixture was stirred with a magnetic stirrer and allowed to stir for one hour at room temperature. Then, 0.375 mmol (362 mg) of tetrakis(bromomethyl)cavitand [16], dissolved in 5.0 mL of dimethyl sulfoxide, was added to the reaction mixture drop by drop. The flask was equipped with a reflux condenser and an argon ball, and the reaction mixture was stirred overnight at 60 °C under an argon atmosphere. The next day, the precipitated solution was cooled to room temperature and poured into 100 mL of 4% hydrochloric acid solution. The filtrate was filtered through a glass filter and washed several times with methanol and dried under vacuum at 80 °C. Pale brown powder (462 mg, 93%). Melting point: >300 °C. ^1^H NMR (500.1 MHz, CDCl_3_): 1.77 (d, *J* = 7.3 Hz, 12H, C*H_3_*CH), 4.11 (d, *J* = 7.3 Hz, 4H, inner OC*H*_2_O), 4.75 (s, 8H, ArC*H_2_*O), 5.01 (q, *J* = 7.0 Hz, 4H, C*H*CH_3_), 5.50 (d, *J* = 7.3 Hz, 4H, outer OC*H*_2_O), and 7.10–7.60 (m, 40H, Ar). ^13^C NMR (125.1 MHz, CDCl_3_): 16.1 (*C*H_3_CH), 31.1 (CH_3_*C*H), 63.6 (Ar*C*H_2_O), 99.7 (O*C*H_2_O), 117.0, 120.4, 122.4, 122.6, 126.8, 127.9, 128.7, 129.5, 131.3, 133.0, 138.8, 153.8, and 156.2.

## 3. Results and Discussion

**Figure 2:**
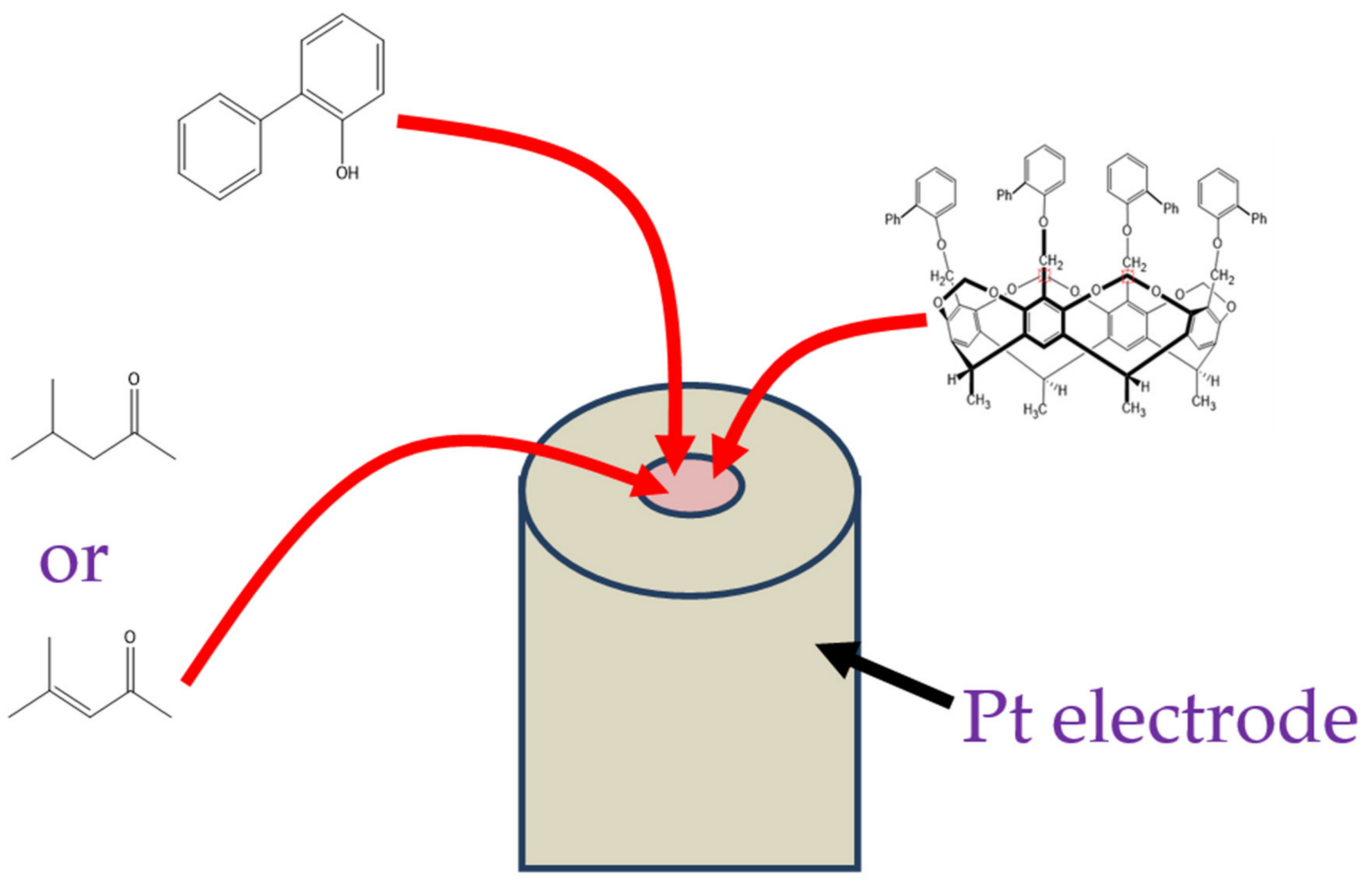
Schematic of the experimental design for the electropolymerization reactions of 2-phenylphenol.

**Figure 3:**
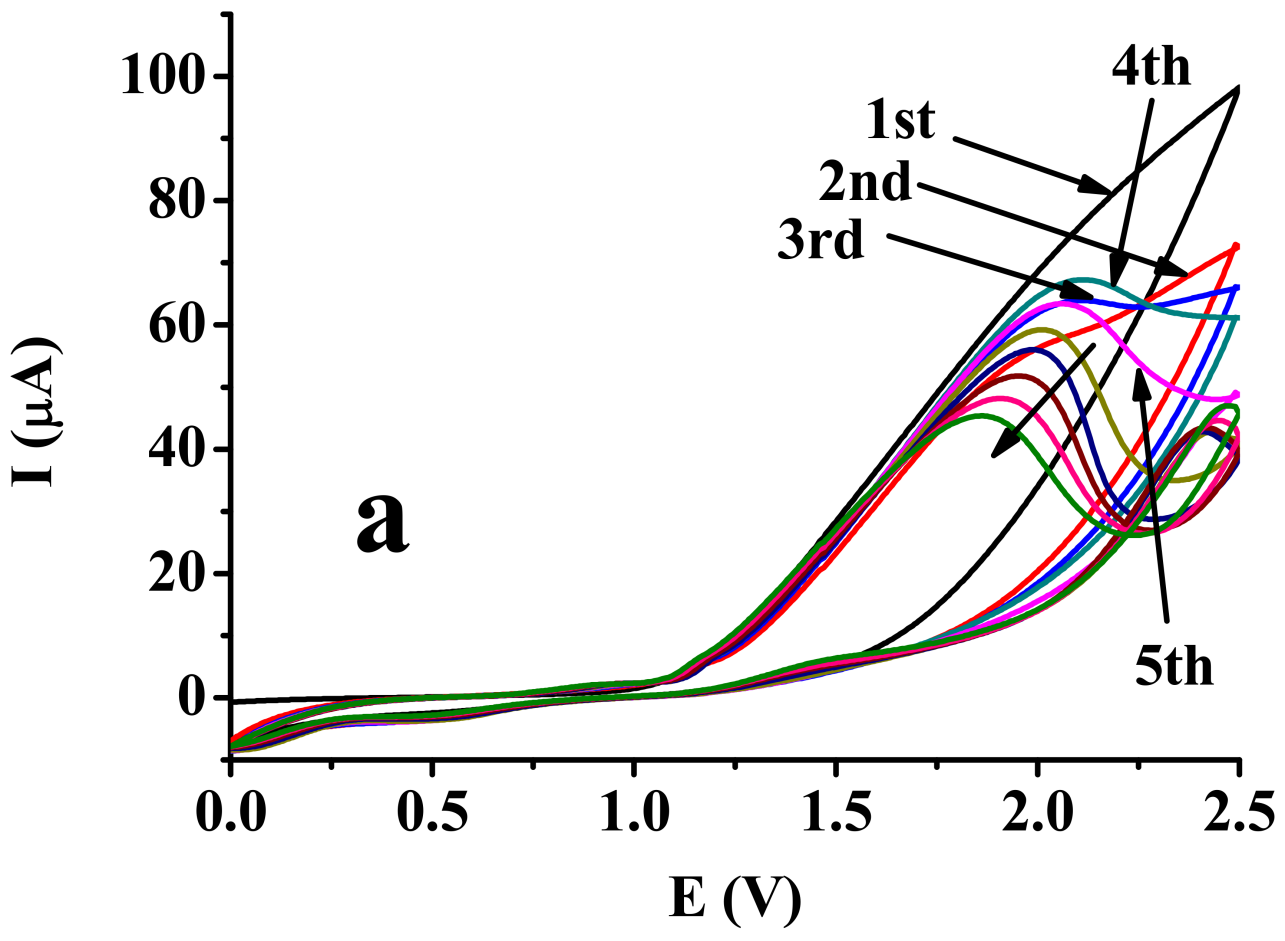

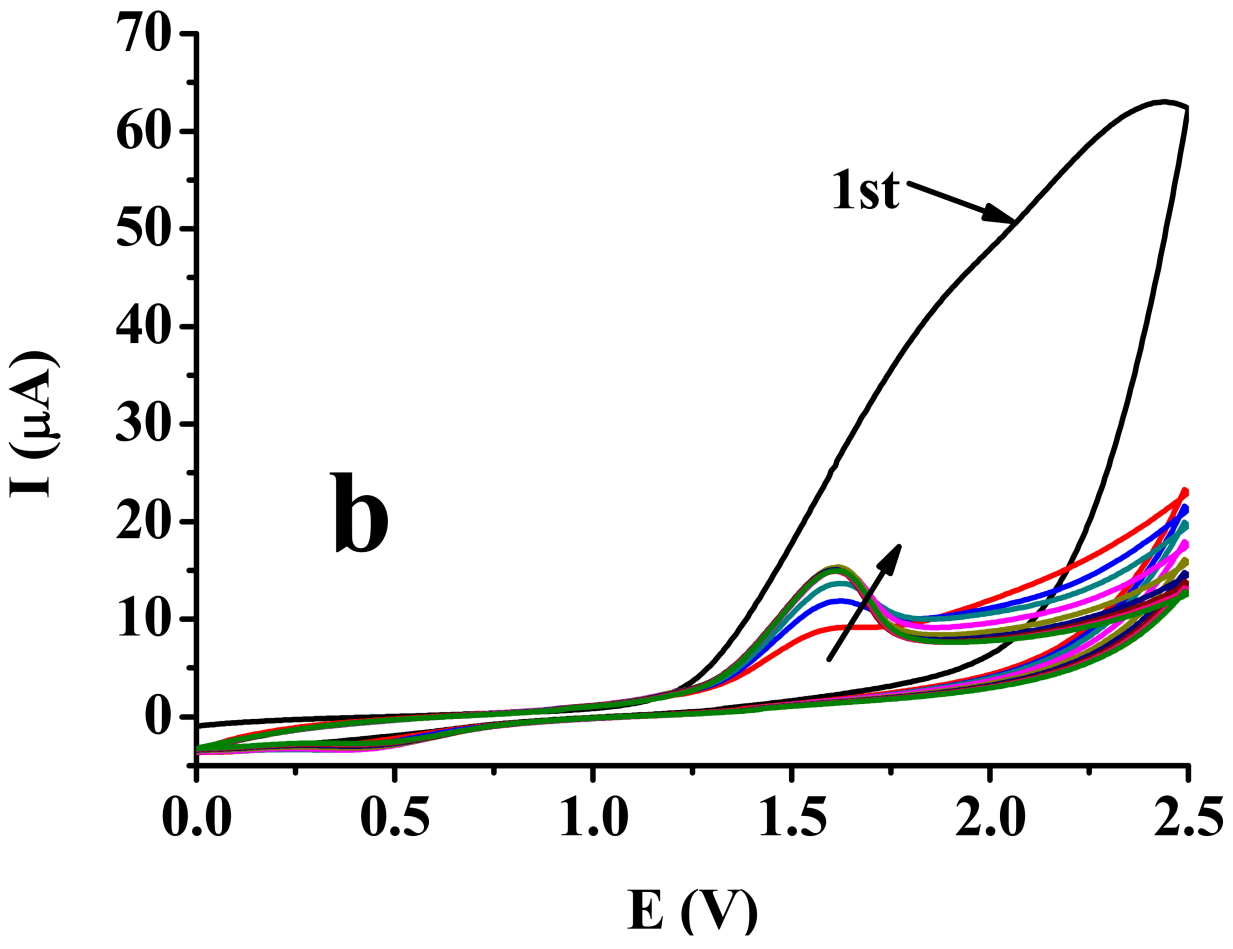

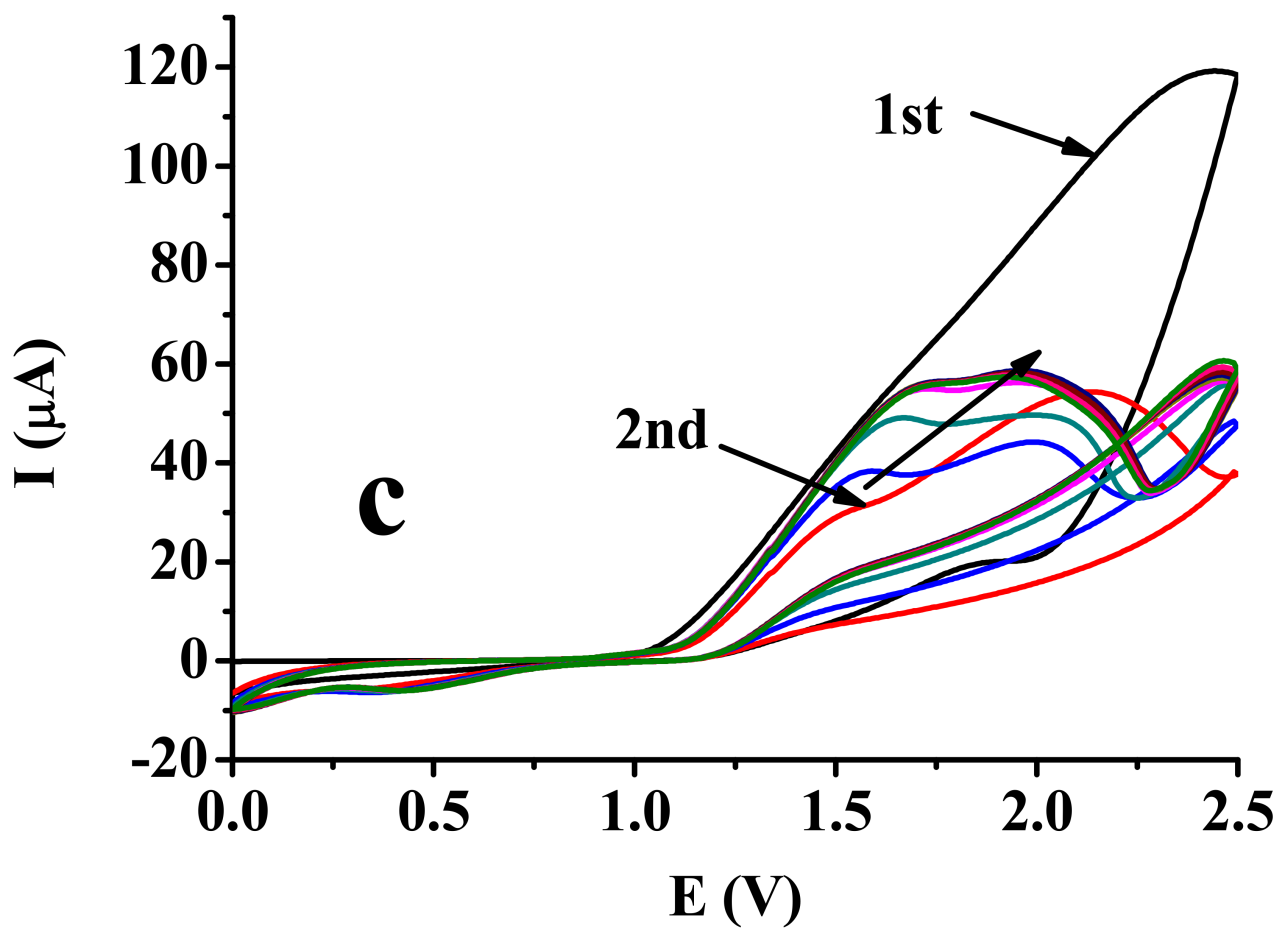
The ten consecutive cyclic voltammograms of 2-phenylphenol (**a**); 3-phenylphenol (**b**); 4-phenylphenol (**c**) in methyl isobutyl ketone (*c* = 50 mM, supporting electrolyte = 70 mM TBAP, and *v* = 0.1 V/s).

A schematic representation of the experimental procedure is shown in Figure 2 showing how the deposition was carried out on the platinum electrode. The solvents methyl isobutyl ketone and mesityl oxide had interesting effects on the electropolymerization reactions; thus, in the first part of our study, the electropolymerization reactions of the three phenylphenol isomers were explored by taking ten subsequent cyclic voltammograms between 0 and 2.5 V with a 0.1 V/s scan rate. In phenylphenols, the neighboring aromatic groups displayed electronic interactions due to the conjugation of the highly delocalized electronic system, which also affected their electrochemical polymerization reactions. When they were investigated, themselves, the differences between isomers could be estimated and a few signs regarding the polymer structures could mostly be found to be related to porosity. A light brown deposit was formed in both solvents from each of the isomers, but the layers deposited from mesityl oxide were thicker. They could easily be removed mechanically from the platinum surface, indicating weak adhesion. The CVs in methyl isobutyl ketone demonstrated deactivation of the platinum electrode in the case of the three phenylphenol isomers (Figure 3). These findings are in accordance with our earlier investigations when phenol was the substrate [14]. This process was very strong (especially remarkable) in the case of 3-phenylphenol. During the entire electropolymerization process, no tendency towards a decrease in the anodic peak currents of the monomers was observed. This was due to the swelling of the organic deposits in methyl isobutyl ketone. However, phenylphenols are bulky substrates due to the phenyl substituents, leading to more open structures of polymers, which is why swelling of their polymers was more pronounced, especially in ketone solvents.

**Figure 4:**
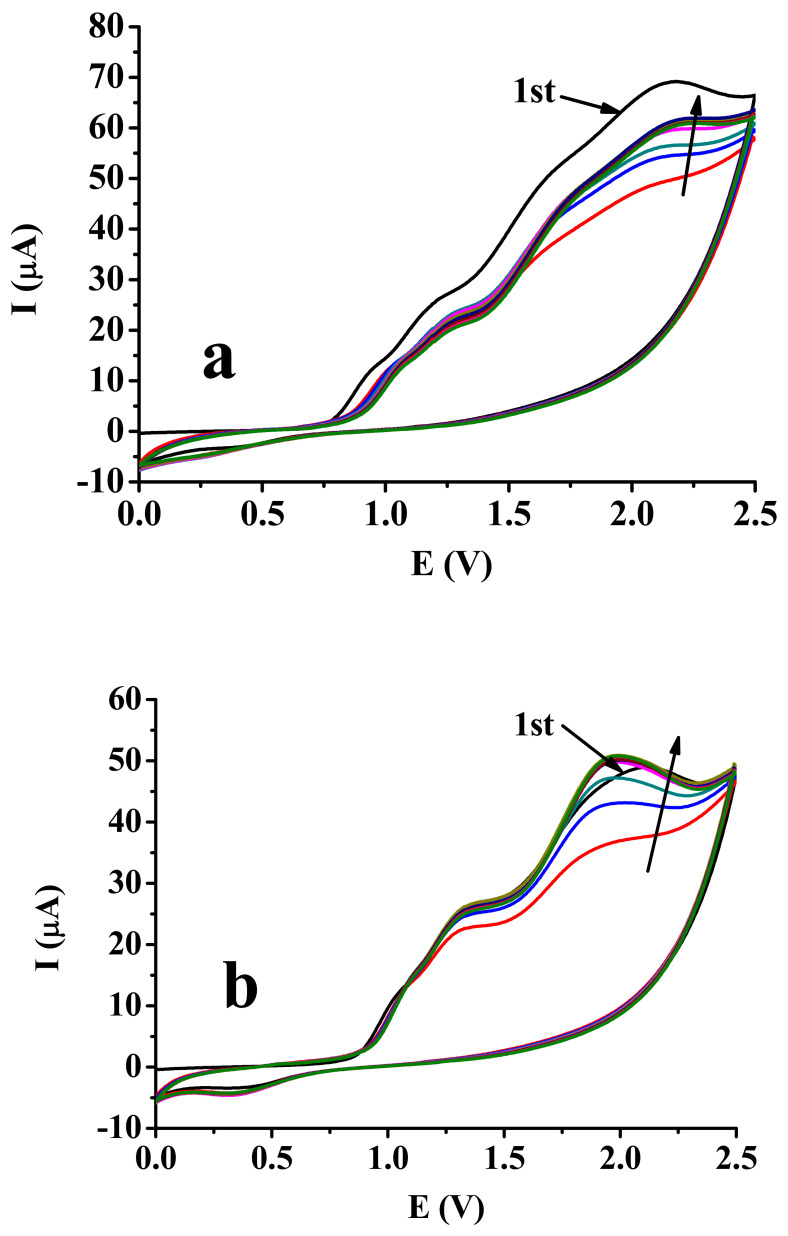

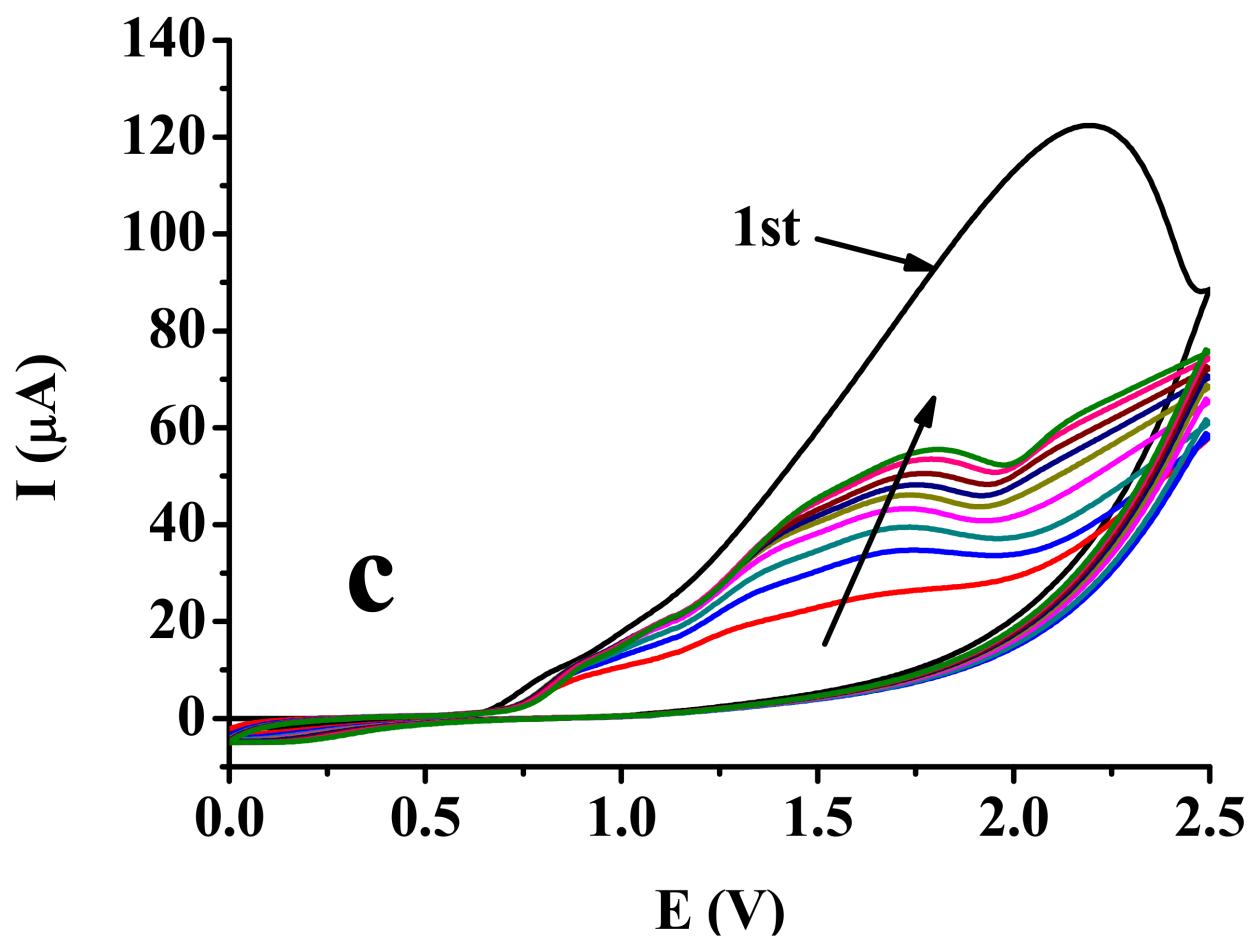
The ten consecutive cyclic voltammograms of 2-phenylphenol (**a**); 3-phenylphenol (**b**); 4-phenylphenol (**c**) in mesityl oxide (*c* = 50 mM, supporting electrolyte = 70 mM TBAP, and *v* = 0.1 V/s).

The differences resulting from methyl isobutyl ketone were also observed for the phenylphenol isomers in mesityl oxide in the electrochemical polymerization process (Figure 4). This solvent had remarkable features compared to the other commonly used nonaqueous solvents, as could be observed. As expected, in the case of 2-phenylphenol and 3-phenylphenol, the drops in the current after the first cycle were not very high, except for 4-phenylphenol. For the latter substrate, a significant swelling was observed in the structure of the polymer, as radicals predominantly joined through the para position of the phenyl substituent. This resulted in the formation of fibrous polymers that could easily attach through weak interactions with neighboring aromatic segments based on *π*–*π* interactions. The structure became obviously more open with the insertion of solvent molecules into the polymer chains.

### 3.1. Electrochemical Polymerizations in the Presence of the Cavitand (***1***)

Although many works can be found that report on the reversible redox behavior of many cavitands bearing functional groups that make them capable of fast electron transfer, some investigations focused on the electrochemistry of cavitands susceptible to electropolymerization. A derivative containing phenol moieties in its cavity exhibited electrode fouling during anodic oxidation [17] and calix[4]arenequinones [18]. Cavitands bearing functional groups with pyrrole moieties provide the possibility for their homopolymerization through their pyrrole moieties [19,20]. In this work, a cavitand was selected that had biphenyl groups at the upper rim attached through an ether bond to the ring skeleton. These aryl ether parts make the molecule electrochemically active through formation of a radical, being then capable of involvement in copolymer synthesis through anodic oxidation. This procedure is different from the simple covering of gold surfaces with cavitands, which is also a promising modifying method [21,22].

**Scheme 1:**
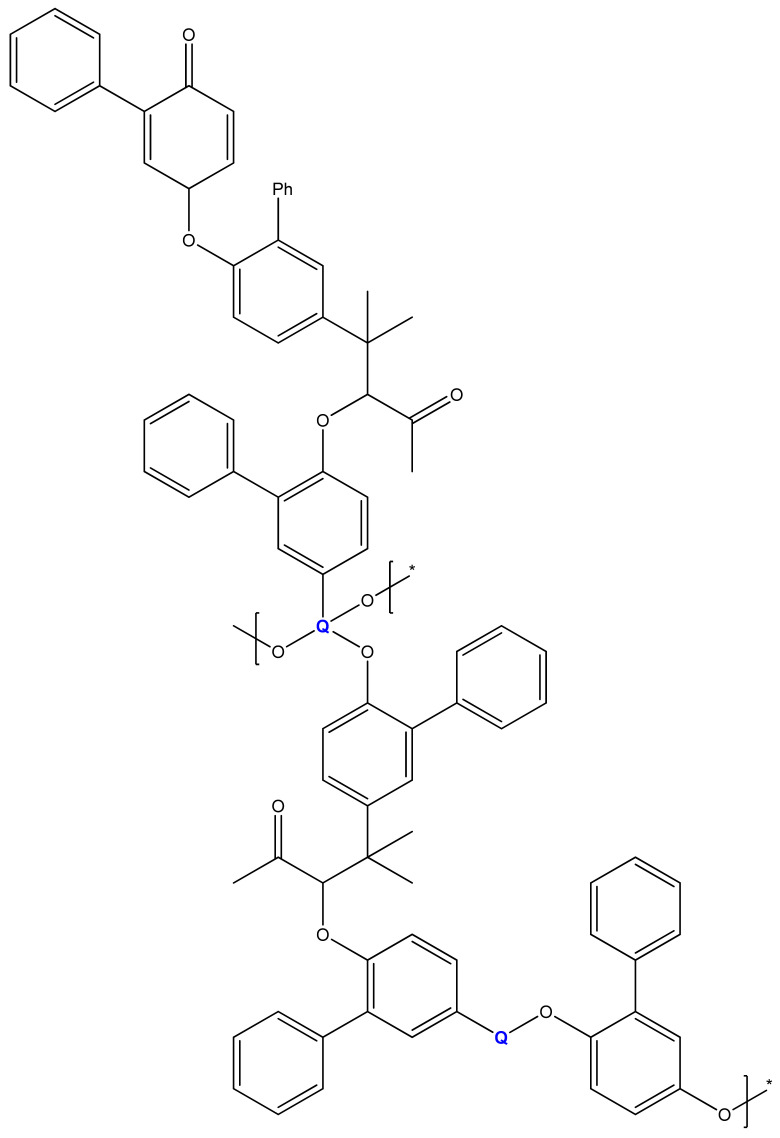
Schematic representation of the coelectropolymerization reaction of 2-phenylphenol with cavitand (**1**) in mesityl oxide (Q: cavitand (1), Ph: phenyl group).

**Figure 5:**
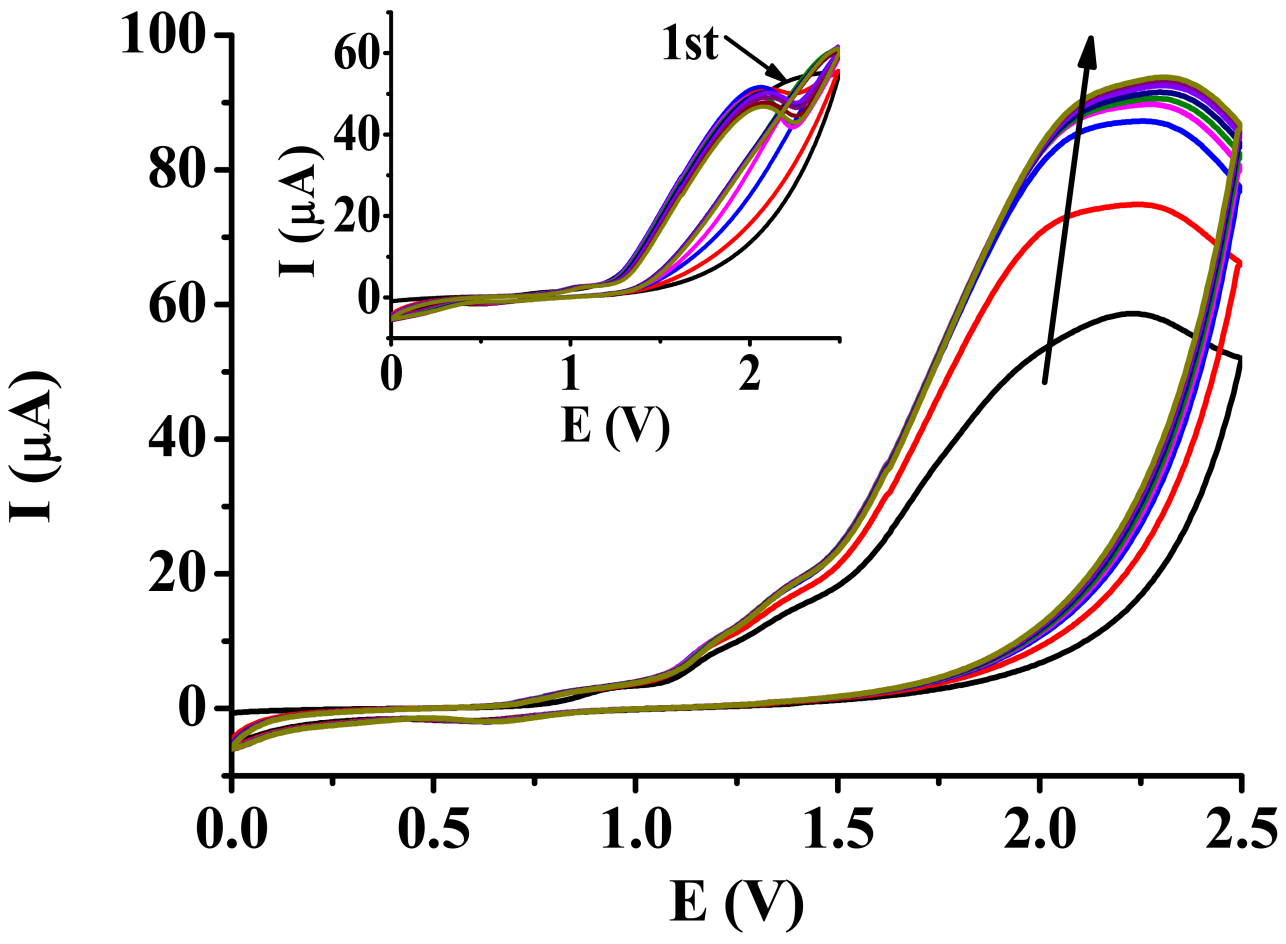
Repetitive cyclic voltammetric curves recorded in a solution prepared with mesityl oxide containing 20 mM 2-phenylphenol and 20 mM cavitand (**1**) (supporting electrolyte = 50 mM TBAP and *v* = 0.1 V/s; inset graph: the same as with methyl isobutyl ketone).

The cavitand chosen can be oxidized on its biphenyl groups due to the fact of its phenylether moiety, which was verified in an indifferent nonaqueous solvent, acetonitrile. It did not show any deactivation, indicating that the biphenyl groups could attach through oxidative dimerization, and the products diverged from the electrode’s surface. The electropolymerization studies were repeated in the presence of the cavitand (**1**) with the phenylphenol isomers in either methyl isobutyl ketone or mesityl oxide, and the concentration of the substrates was 20 mM, respectively. The resultant voltammograms are displayed in Figure 5, and they clearly show that there was a remarkable difference between the two solvents. In methyl isobutyl ketone, the curves were almost reproducible, with a small decline in the current indicating that the involvement of cavitand molecules in the deposit had a modifying effect on the polymer’s structure. This way, the elevated diffusion of electroactive species could be observed. Surprisingly, in mesityl oxide, the anodic peak currents in the subsequent scans increased gradually to a saturation value. Similar behavior was previously observed for phenylether compounds in mesityl oxide [15], but the current enhancement here was more obvious. The presence of cavitand contributed to the development of a more open structure. The formation of the copolymerization product can be rationalized as depicted in Scheme 1. As can be seen, the oxidized biphenyl moieties of the cavitand attached to the growing polymer chain of the poly(phenylphenols). Some of the biphenyl groups or all four of them can be involved in the polymerization process.

**Figure 6:**
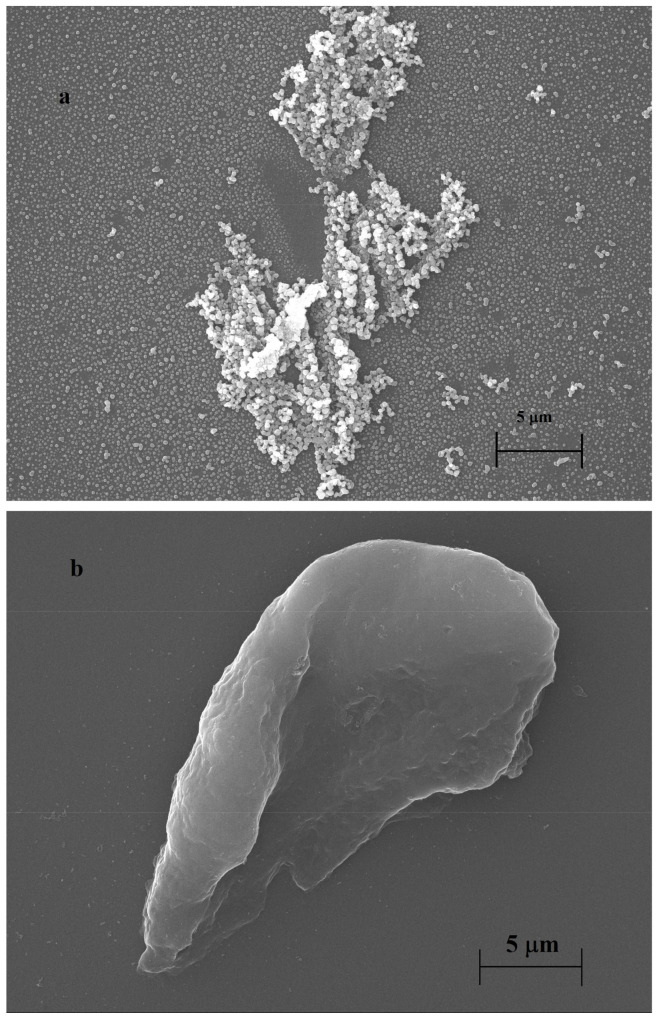

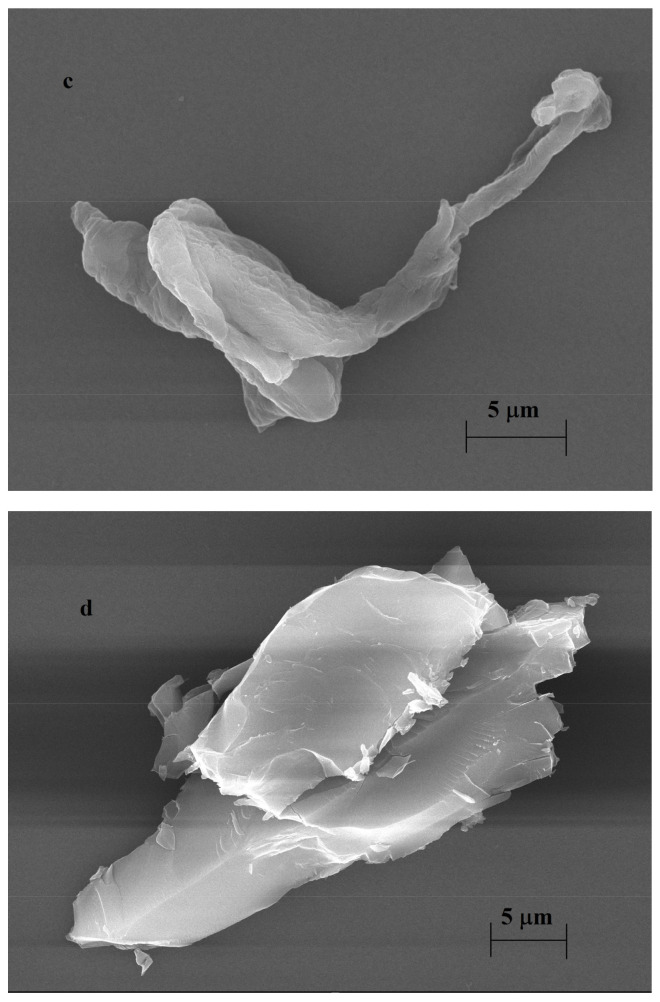
SEM micrographs of deposits of poly(2-phenylphenol) from MIBK (**a**); copolymer of 2-phenylphenol with cavitand (**1**) from MIBK (**b**); poly(2-phenylphenol) from MO (**c**); copolymer of 2-phenylphenol and cavitand (**1**) (**d**) (concentration of the substrates = 20 mM, electrode = ITO-coated glass slide, and acceleration voltage = 30 kV).

The micrographs of the deposit of 2-phenylphenol from MIBK and MO reveal some interesting characteristics, which are displayed in Figure 6. They are also proof demonstrating that thick and coherent films build up in substrate concentrations of approximately 50 mM or higher. Brown deposits could not be seen at 20 mM concentrations by the naked eye. Stackings of electrosynthesized polymers were observed in each case, but there were remarkable differences between the two solvents. Many small spherical-shaped particles formed in methyl isobutyl ketone, but they decreased in number when electrodeposition was carried out in the presence of cavitand (**1**). This is also a sign that the cavitand modifies the electrochemical polymerization of phenylphenols. These small particles could not be seen when the electropolymerization was carried out in MO and the materials were concentrated mainly in higher building blocks.

### 3.2. Analytical Performance of the Prepared Films as a Modifying Layer

**Figure 7:**
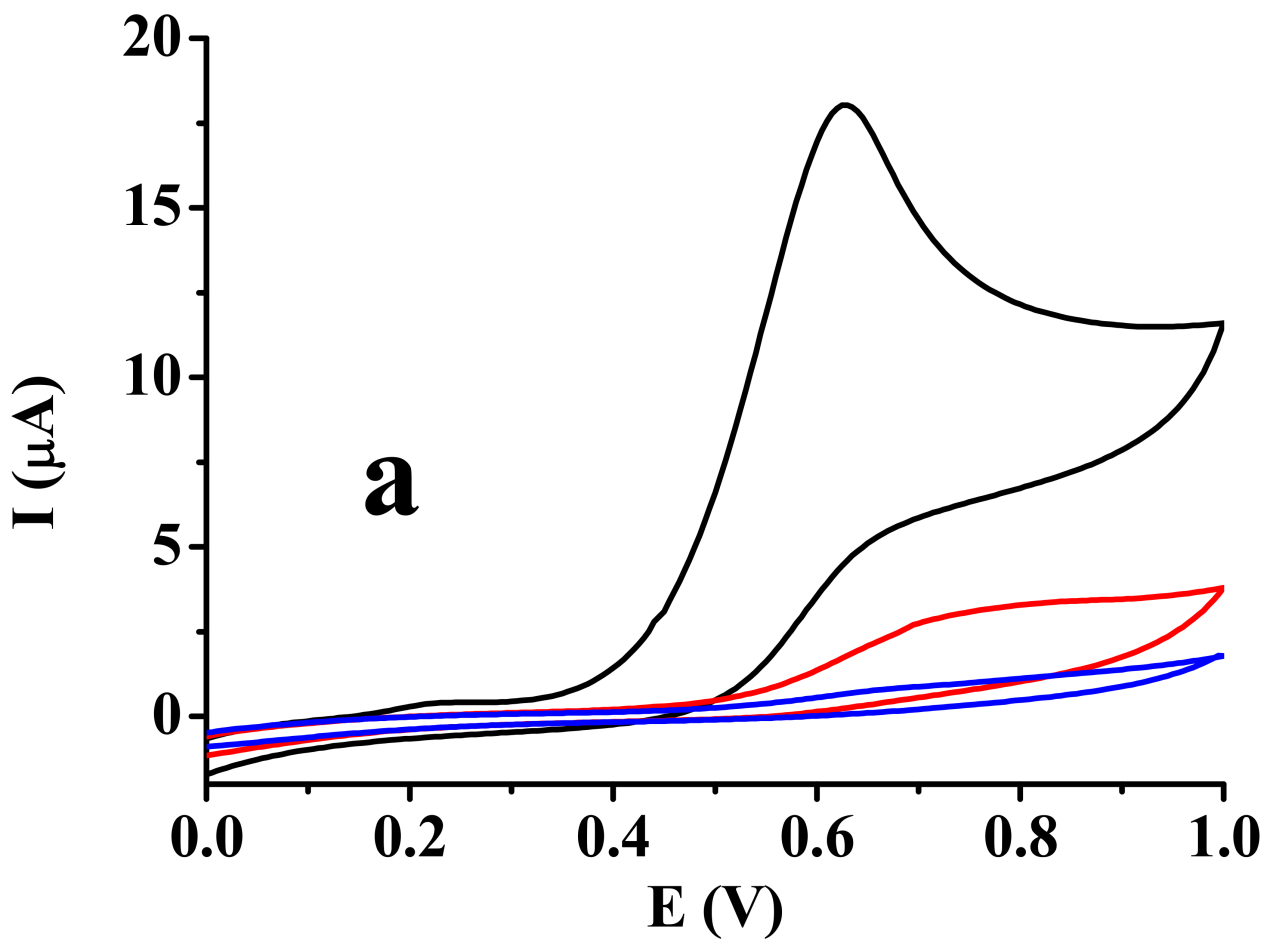

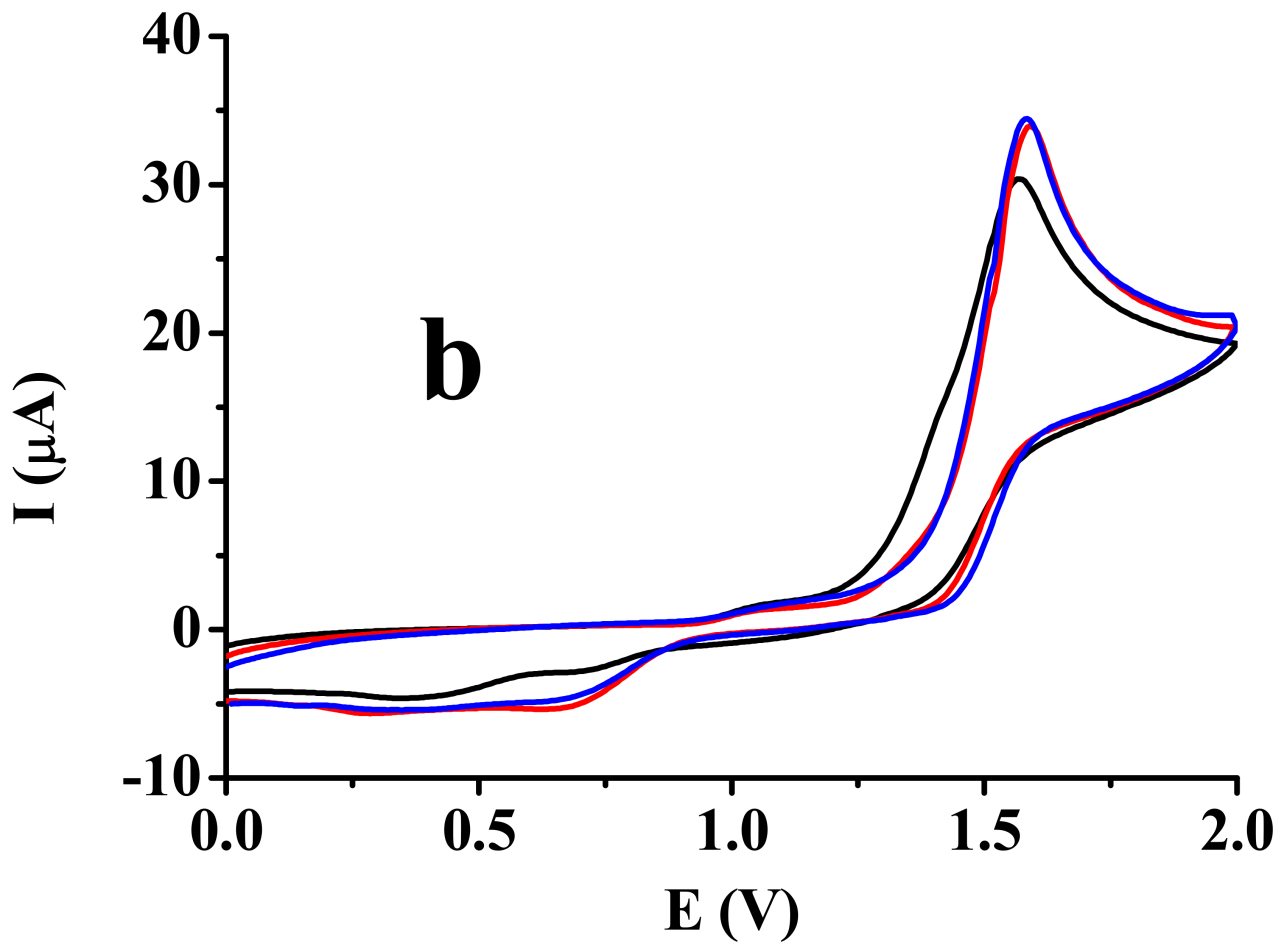
Cyclic voltammograms of 5 mM 4-methoxyphenol in the aqueous phase (**a**) (supporting electrolyte = 0.1 M at pH = 7 in phosphate buffer) and in acetonitrile (**b**) (supporting electrolyte = 20 mM TBAP) (*v* = 0.1 V/s; black curve = bare electrode; red curve = electrode modified with poly(2-phenylphenol); blue curve = electrode modified with poly(2-phenylphenol) copolymer with the cavitand (**1**)).

Many modifying layers can improve the applicability of electrodes in the analysis of selected compounds; therefore, the different deposits were compared in the analysis of 4-chlorophenol and 4-methoxyphenol. Earlier, we found that the oxidation potential of these phenols differed remarkably [23]; thus, they could be detected selectively in their coexistence. As 4-methoxyphenol also has a weak susceptibility to fouling in aqueous environments at concentrations that are not too high, its voltammograms were used for displaying the curves so that the layers could be characterized. However, it is important to mention that 4-chlorophenol also exhibited reproducible voltammograms in nonaqueous solvents up to certain mmol/L concentrations; thus, the effects of the conditions on its analytical signal in such environments will be elevated as well. The results with a modifying layer prepared from 2-phenylphenol and with copolymerization from 2-phenylphenol and cavitand (**1**) in mesityl oxide is displayed (Figure 7).

The voltammetric curves of 4-methoxyphenol taken in an aqueous solution show that with the modified electrodes no anodic peak appeared, and they had a similar shape as was recorded in the background solution (Figure 7a). This suggests that the modifying layers had high hydrophobicity, indicating also that their presence on the platinum was highly disadvantageous. Furthermore, the results indicate that a thin organic layer was present on the surface of the platinum electrode. Contrarily, in the acetonitrile solutions, peaks appeared with the modified electrodes at approximately also the same potential as with the bare electrode (Figure 7b). Moreover, the peak currents were higher for all of the modified electrodes. Comparing the microscopic results with the voltammetric ones, the substrates accumulated on the stackings, causing an increase in the peak currents, attributable partly to cavitand (**1**). The organic layer of 2-phenylphenol, itself, contributed to the increased analytical signal.

By comparing the modifying layers, it is remarkable that there was only a subtle difference between the polymer prepared electrochemically from 2-phenylphenol and the polymer codeposited from 2-phenylphenol and cavitand (**1**). As 2-phenylphenol is able to bind to other molecules through *π*–*π* interactions and, therefore, the resulting polymer with electrooxidation also has aromatic moieties, both of them accommodated readily in the cavity of the cavitand. The latter will be engaged in the prepared layer. As a matter of fact, a segment of the polyphenol oligomer will occupy the inside cavity of the macromolecule. The polymer structure of 2-phenylphenol enables surrounding by multiple aromatic molecules through aromatic segments responsible for signal enhancement.

The above experiments were repeated with the other phenylphenol isomers in mesityl oxide and methyl isobutyl ketone as well. The corresponding data were collected for the two analytes (i.e., 4-methoxyphenol and 4-chlorophenol) in Table 1.

The recovery data show a pronounced difference between the two solvents, as in each case, the layer deposited from methyl isobutyl ketone exhibited a predominant role in hindering the diffusion influencing the magnitude of the analytical signal of the selected phenols. On the contrary, all layers exhibited some accumulation of analytes prepared in mesityl oxide. In the case of polymers prepared in methyl isobutyl ketone, the tortuosity effect predominately contributed to the decline in the current signals, but, of course, analyte molecules can accumulate also at these deposits; however, their majority is far from the electrode.

**Figure 8:**
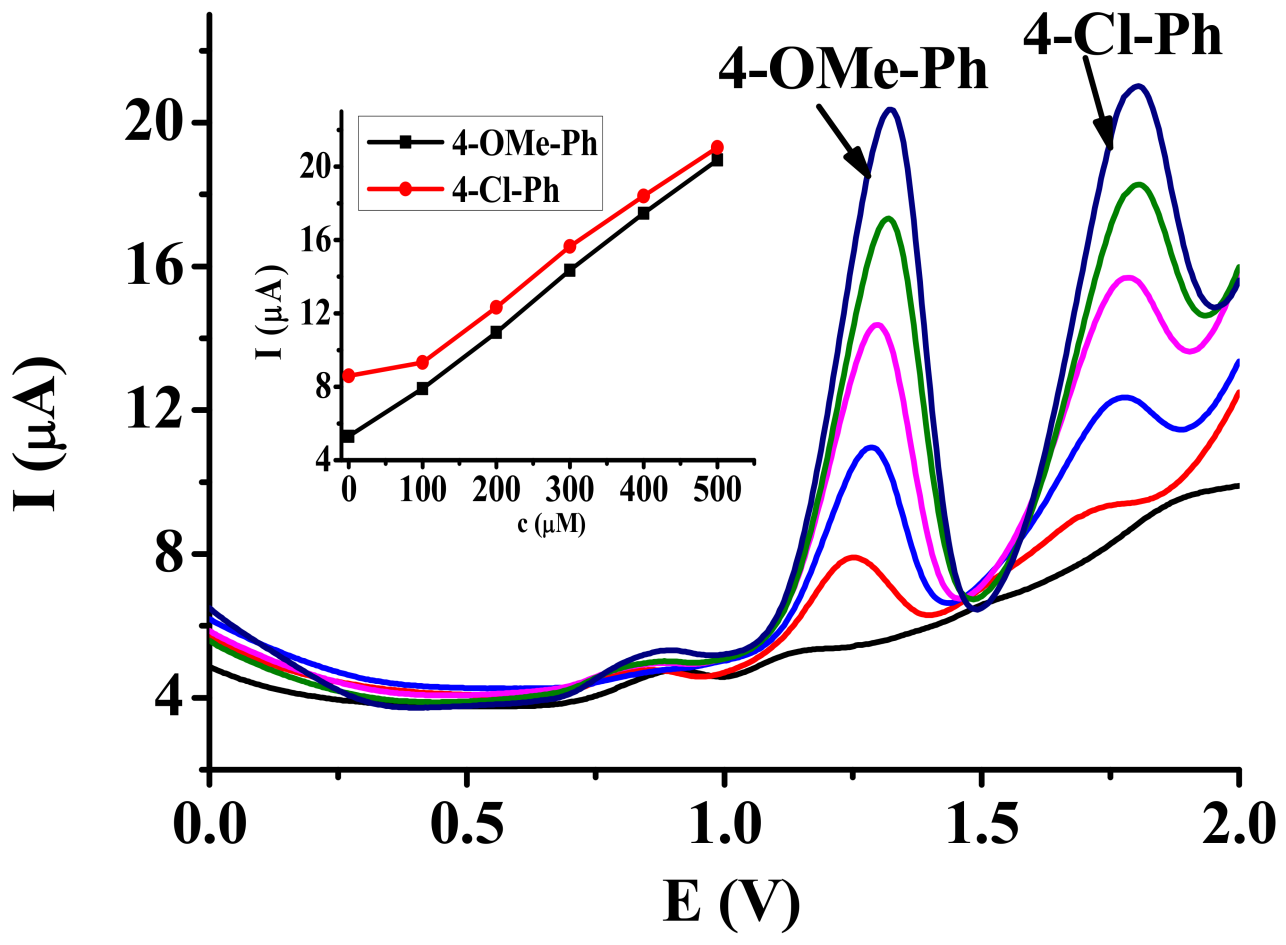
Differential pulse voltammograms of 4-methoxyphenol (4-OMe-Ph) and 4-chlorophenol (4-Cl-Ph) with a modifying layer as the copolymer of 2-phenylphenol and cavitand (**1**) (*E*_step_ = 0.04 V, *E*_pulse_ = 0.25 V, *t*_pulse_ = 5 ms, and *v* = 0.04 V/s; inset graph = calibration curves).

As differential pulse voltammetry provides the possibility for the sensitive determination of compounds, calibration was carried out for the selected phenols (Figure 8). This technique uses potential pulses superposed on the potential scanning, extracting the Faradaic current and minimizing the effect of the condenser current. The latter is due to the continuously changing potential. After optimization of the parameters, the curves were recorded in acetonitrile solutions between 0 and 2 V, where the concentrations ranged from 0 to 500 μM. Under the same conditions, as mentioned earlier, the modifying layer was prepared with ten successive scans with cyclic voltammetry. Before any analytical procedure, additional differential pulse voltammetric measurements had to be carried out with the freshly prepared layer, as the currents closer to 2 V dropped significantly in the first measurements. When the curves did not display a decreasing tendency, the modified electrode was capable for quantification experiments. The curve of 4-methoxyphenol showed a linear dependence in the peak currents over the entire concentration range but only from approximately 100 μM in the case of 4-chlorophenol. The 3*σ*/*S* criterion can be used for 4-methoxyphenol due to the linear dependence; therefore, a 9.28 μM detection limit could be established for it (*S*: slope of the calibration curve; *σ*; scattering of the background signal). On the contrary, the detection limit for 4-chlorophenol could be determined graphically as the crossing point of the straight line of the best fit of the linear stage and horizontal line at *c* = 0 μM. With this method, a detection limit of 50.8 μM was obtained. The reason for this high value was partly due to the high background currents measured at close to 2 V. Lower adsorption capabilities of the layer towards this phenol derivative are shown in Table 1.

## 4. Conclusions

The obtained results showed that electrochemical copolymerization of a cavitand with other monomers enhances the stronger binding of smaller molecules to the modifying layer. The involvement of a cavitand in a polymer film, therefore, contributes to the enhancement of the analytical signals. On the other hand, the discussed polymer layers are useful only in organic solvents; therefore, one of our future plans is to determine more hydrophilic layers with favorable porosity. These findings provide additional proof of the usefulness of mesityl oxide as a solvent in electrodeposition.

## Figures and Tables

**Table 1:** Recoveries in % of 4-methoxyphenol and 4-chlorophenol with the electrodeposited films.

Modifying Layer	4-Methoxyphenol	4-Chlorophenol
poly(2-phenylphenol) MIBK	92.55	83.44
poly(2-phenylphenol) MIBK + CAV (**1**)	98.54	91.21
poly(2-phenylphenol) MO	111.52	100.63
poly(2-phenylphenol) MO + CAV (**1**)	113.37	104.45
poly(3-phenylphenol) MIBK	92.68	86.77
poly(3-phenylphenol) MIBK + CAV (**1**)	92.57	79.24
poly(3-phenylphenol) MO	103.84	99.28
poly(3-phenylphenol) MO + CAV (**1**)	104.57	100.49
poly(4-phenylphenol) MIBK	94.53	87.01
poly(4-phenylphenol) MIBK + CAV (**1**)	90.29	91.66
poly(4-phenylphenol) MO	105.88	100.67
poly(4-phenylphenol) MO + CAV (**1**)	106.03	100.28

## Data Availability

The data presented in this study are available on request from the corresponding author.

## References

[1] Baranowska I., Bijak K. (2013). Voltammetric determination of disinfectants at multiwalled carbon nanotube modified glassy carbon electrode. J. Anal. Chem..

[2] Karimi-Maleh H., Fakude C.T., Mabuba N., Peleyeju G.M. (2019). The determination of 2-phenylphenol in the presence of 4-chlorophenol using nano-Fe_2_O_3_/ionic liquid paste electrode as an electrochemical sensor. J. Colloid Interf. Sci..

[3] Li H., Li J., Meng D., Peng J., Qiao Q., Yang Z., Xu Q., Hu X. (2011). Sodium dodecyl sulfate sensitized electrochemical method for subnanomole level determination of ortho-phenylphenol at a novel disposable electrode. Sci. China Chem..

[4] Jeong H., Choi E., Kang S., Nam K., Jeon S. (2000). Electrochemistry of a urea-functionalized calix[4]diquinone sulfate-anion selective receptor. J. Electroanal. Chem..

[5] Guo D., Wang K., Liu Y. (2008). Selective binding behaviours of *p*-sulfonatocalixarenes in aqueous solution. J. Incl. Phenom. Macrocycl. Chem..

[6] Li Y., Csók Z., Szuroczki P., Kollár L., Kiss L., Kunsági-Máté S. (2013). Fluorescence quenching studies on the interaction of a novel deepened cavitand towards some transition metal ions. Anal. Chim. Acta.

[7] Wang F., Chi C., Yu B., Ye B. (2015). Simultaneous voltammetric determination of dopamine and uric acid based on Langmuir-Blodgett film of calixarene modified glassy carbon electrode. Sensor Actuat. B Chem..

[8] Maity D., Chakraborty A., Gunupuru R., Paul P. (2011). Calix[4]arene based molecular sensors with pyrene as fluoregenic unit: Effect of solvent in ion selectivity and colorimetric determination of fluoride. Inorg. Chim. Acta.

[9] Wang F., Wu Y., Lu K., Ye B. (2013). A simple but highly sensitive and selective calixarene-based voltammetric sensor for serotonin. Electrochim. Acta.

[10] Pailleret A., Arrigan D.W. (2001). Electrochemical oxidation of a tetraester calix[4]arene. Electrochem. Commun..

[11] Skinner A.V., Han S., Balasubramanian R. (2017). Rapid selective colorimetric sensing of polyphosphates by ionic calixarene cavitand interdigitated gold nanoparticles. Sensor Actuat. B Chem..

[12] Vaze V.D., Srivastava A.K. (2007). Electrochemical behaviour of folic acid at calixarene based chemically modified electrodes and its determination by adsorptive stripping voltammetry. Electrochim. Acta.

[13] Liska A., Rosenkranz M., Klíma J., Dunsch L., Lhoták P., Ludvík J. (2014). Formation and proof of stable bi-, tri- and tetraradical polyanions during the electrochemical reduction of *cone*-polynitrocalix[4]arenes. An ESR-UV-vis spectroelectrochemical study. Electrochim. Acta.

[14] Kiss L., Kovács F., Li H., Kiss A., Kunsági-Máté S. (2020). Electrochemical polymerization of phenol on platinum and glassy carbon electrodes in mesityl oxide. Chem. Phys. Lett..

[15] Kiss L., Kovács F., Kunsági-Máté S. (2021). Investigation of anodic behaviour of phenylethers in non-aqueous solvents on platinum and glassy carbon electrodes. J. Iran. Chem. Soc..

[16] Preisz Z., Nagymihály Z., Kollár L., Kálai T., Kunsági-Máté S. (2021). Weak Interactions of the Isomers of Phototrexate and Two Cavitand Derivatives. Int. J. Mol. Sci..

[17] Pailleret A., Herzog G., Arrigan D.W. (2003). Electrochemical activity of phenolic calixarenes. Electrochem. Commun..

[18] Meddeb-Limem S., Besbes-Hentati S., Said H., Bouvet M. (2011). From the electrochemical generation of calix[4]arenedihydroquinone to the electrodeposition of calix[4]arenediquinone-calix[4]arenedihydroquinone assembly. Electrochim. Acta.

[19] Buffenoir A., Bidan G., Chalumeau L., Soury-Lavergne I. (1998). Immobilisation at an electrode surface of calix[4]arenes bearing electropolymerisable pyrrole moieties at the upper rim. J. Electroanal. Chem..

[20] Chen Z., Gale P.A., Beer P.D. (1995). Synthesis and electropolymerization of calix[4]arenes containing *N*-substituted pyrrole moieties. J. Electroanal. Chem..

[21] Collyer S.D., Davis F., Lucke A., Stirling CJ M., Higson SP J. (2003). The electrochemistry of the ferri/ferrocyanide couple at a calix[4]resorcinarenetetrathiol-modified gold electrode as a study of novel electrode modifying coatings for use within electro-analytical sensors. J. Electroanal. Chem..

[22] Pan G., Wan L., Zheng Q., Bai C., Itaya K. (2002). Self-organized arrays of calix[4]arene and calix[4]arene diquinone disulphide on Au(111). Chem. Phys. Lett..

[23] Kiss L., Kunsági-Máté S. (2021). Effect of anodic pretreatment on the performance of glassy carbon electrode in acetonitrile and electrooxidation of para-substituted phenols in acetonitrile on platinum and glassy carbon electrode. Period. Polytech. Chem..

